# Cervical cytological changes in HIV-infected patients attending care and treatment clinic at Muhimbili National Hospital, Dar es Salaam, Tanzania

**DOI:** 10.1186/1750-9378-7-3

**Published:** 2012-02-15

**Authors:** Amos R Mwakigonja, Liset Maria M Torres, Henry A Mwakyoma, Ephata E Kaaya

**Affiliations:** 1 Department of Pathology, Muhimbili University of Health and Allied Sciences (MUHAS), P.O. Box 65001, Dar es Salaam, Tanzania

## Abstract

**Background:**

Tanzania is among Sub-Saharan countries mostly affected by the HIV and AIDS pandemic, females being more vulnerable than males. HIV infected women appear to have a higher rate of persistent infection by high risk types of human papillomavirus (HPV) strongly associated with high-grade squamous intraepithelial lesions (HSIL) and invasive cervical carcinoma. Furthermore, although HIV infection and cervical cancer are major public health problems, the frequency and HIV/HPV association of cervical cancer and HSIL is not well documented in Tanzania, thus limiting the development of preventive and therapeutic strategies.

**Methods:**

A prospective unmatched, case-control study of HIV-seropositive, ≥ 18 years of age and consenting non-pregnant patients attending the care and treatment center (CTC) at Muhimbili National Hoospital (MNH) as cases was done between 2005 and 2006. HIV seronegative, non-pregnant and consenting women recruited from the Cervical Cancer Screening unit (CCSU) at ORCI were used as controls while those who did not consent to study participation and/or individuals under < 18 years were excluded. Pap smears were collected for routine cytodiagnosis and P53 immunohistochemistry (IHC). Cervical lesions were classified according to the Modified Bethesda System.

**Results:**

A total of 170 participants from the two centers were recruited including 50 HIV-seronegative controls were from the CCSU. Ages ranged from 20-66 years (mean 40.5 years) for cases and 20-69 years (mean 41.6 years) for controls. The age group 36-45 years was the most affected by HIV (39.2%, n = 47). Cervicitis, squamous intraepithelial lesions (SIL) and carcinoma constituted 28.3% (n = 34), 38.3% (n = 46) and 5.8% (n = 7) respectively among cases, and 28% (n = 14), 34% (n = 17) and 2% (n = 1) for controls, although this was not statistically significant (P-value = 0.61). IHC showed that p53 was not detectable in HPV + Pap smears and cell blocks indicating possible degradation.

**Conclusions:**

The frequency of SIL and carcinoma appeared to be higher among HIV-infected women on HAART compared to seronegative controls and as expected increased with age. HIV seropositive patients appeared to present earlier with SIL compared to those HIV seronegative suggesting a role of HIV in altering the natural history of HPV infection and cervical lesions. The absence of p53 immunoreactivity in HPV + lesions is indicative of the ability of HPV E6 proteins to interact with the tumor suppressor gene and pave way for viral-induced oncogenesis in the studied Tanzanian women.

## Background

Cancer of the uterine cervix remains the second commonest cancer among women worldwide [1], more than 85% of the global burden of this disease occurs in the developing world [1] being the commonest malignancy in women in Sub-Saharan Africa according to Globocan 2008 (IARC) [2]. The introduction of the Papanicolaou (Pap) smear in 1930's made early detection and treatment of pre-invasive disease possible, which has significantly reduced morbidity and mortality related to cervical cancer in developed but not developing countries, due to the absence of effective screening programs [3]. HIV-infected women have a higher likelihood of developing persistent high-risk human papillomavirus (HPV) infection, precancer [high-grade squamous intraepithelial lesions (HSIL)], and invasive cervical cancer than seronegative women but this has not been clearly documented in Tanzania [1,4]. Furthermore, HIV-associated cervical lesions are multicentric, aggressive and recurrent after treatment [5]. Co-infection with HPV and HIV is to be expected and recent epidemiological data from Africa show that cervical cancer is the most common AIDS defining neoplasm in women [5]. Among women infected with HIV there is a high prevalence of HPV infection [6]. Unlike other AIDS defining neoplasms, the occurrence of cervical cancer is not dependent on immune compromise [5]. It is established that invasive cervical cancer is the end result of progressive changes, beginning with precursor lesions namely cervical dysplasia or cervical intraepithelial neoplasia (CIN) also called squamous intraepithelial lesion (SIL), carcinoma in-situ (CIS) and lastly invasive carcinoma [7]. However, if early detection is made, pre-malignant lesions are reversible and cure of early-stage cancer is achievable. A number of risk factors that contribute to the development of cervical cancer, including low socioeconomic class, sexual intercourse at an early age, multiple sexual partners, multiparity, long-term oral contraceptive use, tobacco smoking, vitamin deficiency and sexually transmitted infection (STI) including *Chlamydia trachomatis *and *Herpes virus type II *have been identified [8-10] including the *human papilloma virus *(HPV) as a primary sexually transmitted etiologic agent in the development of cervical squamous cell carcinoma (CSCC) and its precursors [11]. Furthermore, HIV-related immunosuppression seems to contribute to increased frequency of SIL and HSIL as previously reported from Tanzania although that study did not include invasive cancer [12]. HIV infection and cervical cancer are major public health problems among women in Tanzania, although, the frequency of cervical cancer and pre-cancerous lesions in the general compared to the HIV-infected populations in Tanzania is not well documented and is elucidated in the current study.

## Methods

### Study area

The study was conducted in the Unit of Histopathology/Morbid Anatomy as well as the Department Obstetrics/Gynaecology at the Muhimbili National Hospital (MNH), Dar es Salaam, Tanzania in collaboration with the Cervical Cancer Screening Unit (CCSU) at the Ocean Road Cancer Institute (ORCI).

### Study design and sampling criteria

This was a prospective unmatched, case-control study of HIV-seropositive, ≥ 18 years of age and consenting non-pregnant patients attending the care and treatment center (CTC) for highly active antiretroviral therapy (HAART) at MNH as cases was done between 2005 and 2006. HIV seronegative, non-pregnant and consenting women recruited from the Cervical Cancer Screening unit (CCSU) at ORCI were controls while those who did not consent to study participation and/or individuals under < 18 years were excluded. Thus all women meeting these criteria at the two centres during the study period were included the sample prospectively.

### Laboratory methods

#### HIV serology

HIV-1 evaluation was done at the Department of Microbiology/Immunology, Muhimbili University of Health and Allied Sciences (MUHAS) and was performed on sera as previously described [13,14] and results obtained through the medical records departments of MNH and ORCI.

#### Pap smears

Pap smears were performed by a Gynaecologist at the MNH and ORCI clinics. Patients were put in lithotomy position and a bivalve speculum was introduced. The cervix was exposed under illumination for assessment. Specimens were taken from the squamo-columnar junction using an Ayres spatula. The specimens were smeared on five SuperFrost^® ^glass slides (Menzel GmbH & Co KG, Braunschweigh, Germany) two of them fixed immediately in 95% alcohol/ether for Papanicolaou staining (see below) and the rest air dried for two hours for immunocytochemistry. Slides were examined under a light microscope and cytodiagnosis done by a Pathologist.

#### Papanicolau staining procedure

Alcohol/ether-fixed smears were hydrated in graded alcohol as previously described [15] and stained with Harris Haematoxylin for 5 minutes, differentiated in 1.0% acetic acid in alcohol and blued in running tap water for 3 minutes. Smears were then dehydrated in ascending graded alcohol from 70% to 95% and stained with orange G for 3 minutes, rinsed in 95% alcohol followed by staining with EA 36 for three minutes and then dehydrated in absolute alcohol. Thereafter the smears were placed in xylene to remove alcohol and finally were mounted with DPX and glass cover slips were applied.

#### Immunocytochemistry of acetone fixed smears

The smears were fixed in cold acetone at 4°C for 10 minutes and dried at room temperature (RT) for 10 minutes. After Tris-Buffered Saline (TBS) rehydration, blocking of endogenous peroxidase activity with hydrogen peroxide (H2O2) was done for 30 minutes at RT. Normal Horse Serum (NHS) diluted at 1:20 was then applied for 30 minutes at RT. The excess NHS was wiped off and slides incubated over night at 4°C with primary antibodies [mouse monoclonal anti-human p53 (clone DO-7) (Dako, Glostrup, Denmark)]. After TBS washing slides were then processed and mounted as previously described [15,16].

#### Control of results

Negative controls included sections which did not express the antigen under study and TBS instead of the primary antibody. Positive controls consisted of sections known to express the antigen under investigation.

#### Immunohistochemistry on paraffin control sections

This was done as previously described [15,16]. Five microns (5 μ) thick sections were placed in xylene over night for deparafinization, and hydrated in descending grades of ethanol to distilled water. Antigen retrieval was achieved by heating sections to boiling in Citrate Buffer at pH 6.0 in a microwave oven for 10 minutes and sections then allowed to cool to RT. The remainder of the procedure was done as described above and previously [15,16].

#### Microscopy

The smears were finally evaluated in a Carl Zeiss Axiomatic microscope (Jena, Germany) and digital microphotography was done using an Olympus Camera Model DP 12 (Olympus Corporation, Japan) and pictures processed using Adobe Photoshop 7.0 (Adobe Systems Incorporated, San Jose, USA).

### Data collection and management

Patients' identification, biodata and experimental results were entered in structured and computer coded questionnaires.

### Data analysis

Data analysis was done using computer package; EPI INFO 6 (CDC, Atlanta, Georgia, USA). P-values ≤ 0.05 was considered statistically significant.

### Ethical issues

Ethical permission was sought from the MUHAS Ethical Clearance Committee. Informed consent was sought from patients to screen them for HIV and for cervical cancer. Study participation/non-participation had no bearing whatsoever on the quantity and/or quality of the services the women expected to receive. All study information was processed in confidence and patients' IDs were never exposed.

### Study limitations

a) Cytological changes suggestive of HPV infection may not reflect the true magnitude of HPV infection, since molecular techniques to detect HPV were not done due to logistical constrains.

b) Since only few cases were selected for p53 analysis, the conclusion may not reflect the true extent of p53 inactivation by HPV.

## Results

### Demographic data of the patients

**Figure 1:**
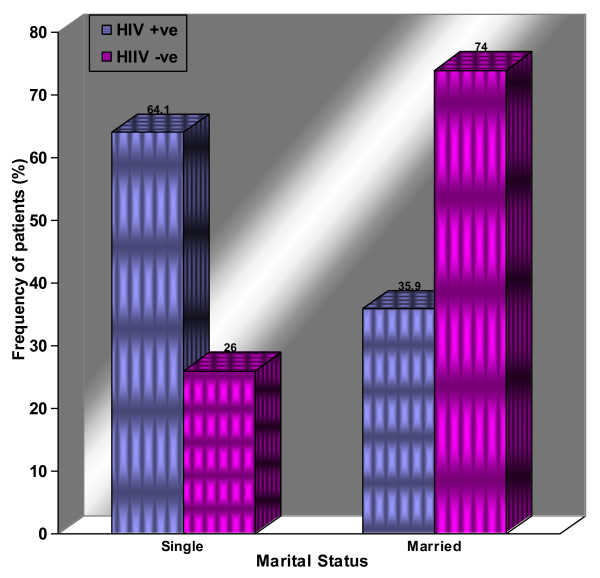
**The distribution of patients according to marital status and HIV infection**. Histograms showing the distribution of patients according to marital status and HIV infection.

A total of 170 women were recruited from the two centers (120 HIV-seropositive cases on HAART from MNH and 50 seronegative controls from ORCI) between 2005 and 2006. Singles constituted 64.1% (77/120) of cases and only 26% (13/50) of controls. The rest of women in each group were married which difference was highly statistically significant (P-value < 0.001), suggesting that singles were more likely to be HIV-infected (Figure 1 and Table 1). Age ranged from 20-66 years (mean 40.5 years) among cases and from 20-69 years (mean 41.6 years) for Controls. The age group of 36-45 years was the most affected (39.2%, 47/120) by HIV infection (Table 1).

**Table 1:** List of the general characteristics of the studied Tanzanian women

Characteristics	Cases	Controls	Combined	P-Value
HIV serostatus	positive	negative		
Age-Range (years)	20-66	20-69	20-69	
Mean Age (years)	40.5	41.6	41.05	
Peak age-group (20-45years) [No (%)]	84 (70.0)	33 (66.0)	117 (68.8)	0.700
Single [No (%)]	77 (64.1)	13 (26.0)	90 (52.9)	< 0.001
Married [No (%)]	43 (35.8)	37 (74.0)	80 (47.1)	< 0.001
Total number	120	50	170	

This table shows that HIV infection was more frequent among singles compared to married women and as expected, majority of HIV seropositive women came from the most sexually active age-group of 20-45 years

### Distribution of cervical lesions

**Figure 2:**
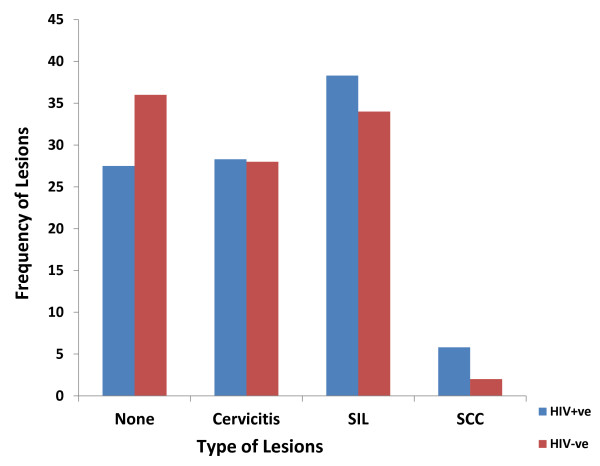
**The distribution of cytological types of cervical carcinoma and HIV status**. This Histogram shows that squamous cell carcinoma (SCC) as well as squamous intraepithelial lesions (SIL) were more common amongst HIV seropositive women.

**Figure 3:**
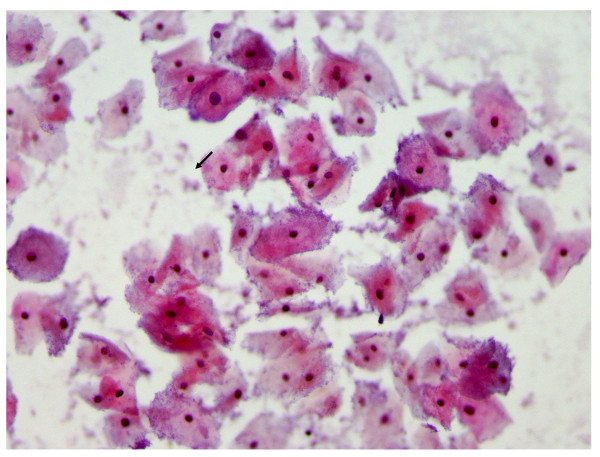
**Pap smear showing bacterial vaginosis**. A micrograph showing Pap staining showing abundant clue cells (arrow) suggestive of bacteria vaginosis more likely due to *Gardnerella vaginalis *infection (× 40).

**Figure 4:**
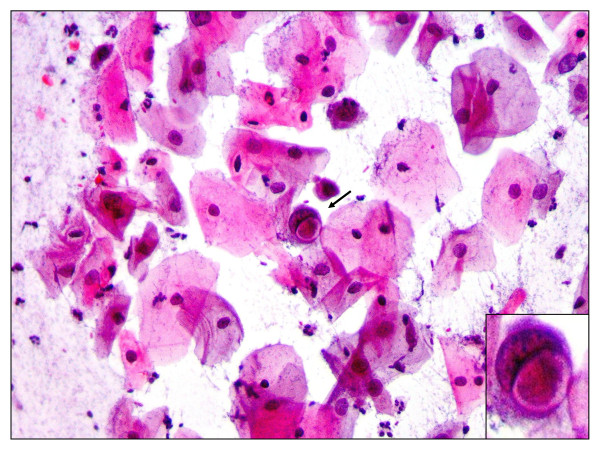
**Pap smear showing Chlamydia cervicitis**. Pap staining in a patient with acute cervicitis showing a squamous cell with cytopathic changes characterized by the presence of a cytoplasmic inclusion body (arrow and inset) indicating Chlamydia infection (× 40).

Cervicitis, squamous intraepithelial lesions (SIL) and carcinoma constituted 28.3% (34/120), 38.3% (46/120) and 5.8% (7/120) among cases, and 28% (14/50), 34% (17/50) and 2% (1/50) for controls respectively, (P-value = 0.6) [Figure 2 and Table 2]. A great majority (94.1%) of cases with cervicitis (32/34) had acute cervicitis [bacterial vaginosis (Figure 3)] and only 2 (5.9%) had chronic inflammation, and all HIV negative controls presented with acute cervicitis (Table 2). In this study 9.4% of smears of HIV infected patients with cervicitis showed some cells with cytoplasmic inclusions, suggesting Chlamydia infection (Figure 4). Furthermore, follicular cervicitis was identified in two conventional smears of HIV infected patients. A great majority (94.1%) of cases with cervicitis (32/34) were single, while a lesser majority (64.3%, 9/14) HIV negative controls were single (P-value = 0.02), indicating that HIV infected patients on HAART who are single were at an increased risk for cervicitis compared with corresponding controls. The same applied to the association of SIL and cervical cancer with marital status, number of sexual partners and increased parity but not with the use of oral contraceptives (OC) were majority of cases and controls with the conditions were non-OC users.

**Table 2:** Distribution of cervicitis and SIL according to HIV serostatus

*HIV status*	*LSIL* *No (%)*	*HSIL* *No (%)*	*Total SIL No (%)*	*P-Values*	*Acute Cervicitis No (%)*	*Chronic Cervicitis No (%)*	*Total Cervicitis No (%)*	*Total Cervical Lesions*	*P-Values*
HIV +ve	27(58.6)	19(41.3)	46 (38.3)	0.25	32(94.1)	2(5.9)	34(28.3)	80(66.7)	P = 0.61
HIV -ve	13(76,4)	4(23.5)	17(34.0)	0.25	14(100.0)	0(0.0)	14 (28.0)	31(62.0)	P = 0.61
**Total**	**40 (63.5)**	**23(36.5)**	**63(56.8)**	0.25	**46(95.8)**	**2(4.2)**	**48(43.2)**	**111(100.0)**	P = 0.61

This table shows that the distribution of cervicitis did not vary with HIV serostatus and the distribution of HSIL appeared more likely to vary with HIV serostatus compared to LSIL although both were not statistically significant

**Figure 5:**
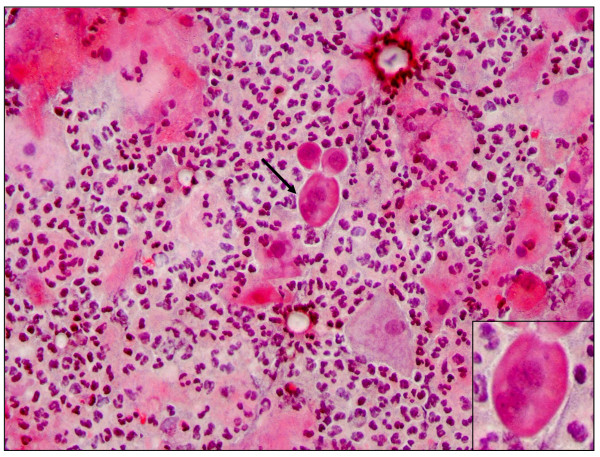
**Pap smear showing LSIL**. Pap staining in a patient with LSIL showing extensive leukocytic background and a binucleated parabasal squamous cell with mild nuclear enlargement and fine granular chromatin (arrow and inset) suggestive of viral (HPV) or TV infection (× 100).

**Figure 6:**
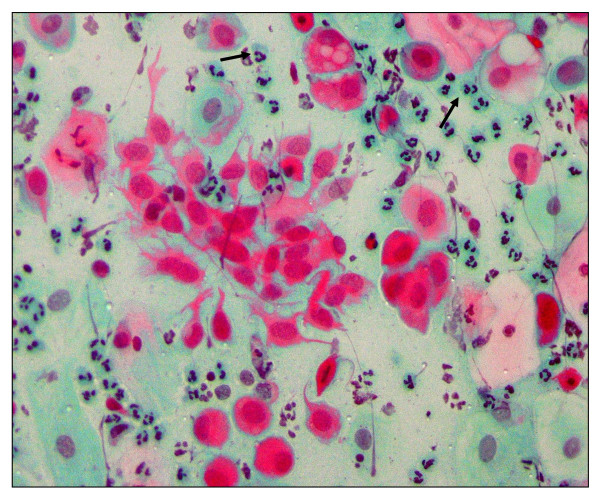
**Pap smear showing HSIL**. Pap staining in a patient with HSIL showing abundant neutrophils, a cluster of dendritic cells. The arrows show a parabasal squamous cell with multiple cytoplasmic vacuolation suggestive of viral infection (× 40).

**Figure 7:**
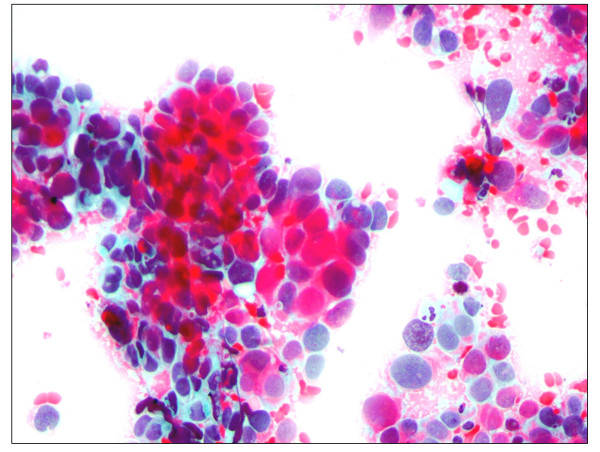
**Pap smear showing cervical cancer**. Pap staining in a patient with squamous carcinoma which exhibits clusters of cohesive cells with marked nuclear enlargement, pleomorphisms, hyperchromatism and keratinization (× 40).

Majority (58.6%, 27/46) of SIL were LSIL (Figure 5) and the rest were HSIL (Figure 6). However, a greater majority (76.4%) of HIV negative controls had LSIL (13/17) (P-value = 0.25) [Table 2]. Binucleated cells suggestive of HPV or TV infections were present on 18.5% (5/27) smears of HIV infected patients on HAART and 7.7% (1/13) smears of HIV negative control (Figure 5). A great majority (85.7%, 6/7) of cases with carcinoma had squamous cell carcinoma (SCC) but 1 (14.3%) had adenocarcinoma (AC) [Figure 7]. Only one patient with cervical malignancy amongst controls had SCC. Orangeophilic cells suggestive of keratinization, were found in 66.6% (4/6) cases (Figure 7). The distribution of cytological features suggestive of HPV infection (koilocytosis) between cases and controls was not statistically significant (P-value = 0.76). However, this was more frequent (77.8%, 21/27) in smears of cases older than 35 years but less so (55.6%, 5/9) of smears from controls. Furthermore, a great majority (96.3%, 26/27) of cases with cervical koilocytosis had had first sexual intercourse before age 17 years but less so (77.8%, 7/9), among controls (P-value = 0.02). This implies that an early age of sexual debut increases the risk to HIV, HPV and/or other STI.

### Immunohistochemistry

This was performed on cell blocks and smears from the cervix fixed in cold acetone for 13 cases of LSIL, 7 cases of HSIL and 4 cases of carcinoma with cytomorphological features suggestive of HPV infection. The expression of p53 was absent in the tested slides compared with the colorectal carcinoma used as positive control, in which the expression of p53 was strong and nuclear in location. These results suggest the ability of HPV oncogenes (like E6) to interact with p53 although the samples tested were few.

## Discussion

The increased frequency of cervical lesions generally among adults in this study contrasted those of a previous study reporting a peak among adolescents and young adults [17]. However, the discrepancy in the results could be due to selection bias considering that all our patients were recruited from the CTC, which is mainly attended by patients older than 25 years on ART and hence not representative of the general population.

In the present study the frequency of cervicitis among HIV infected patients appears higher compared to a US study where it was reported to be 15% [18]. The difference may be due to the higher prevalence of HIV and AIDS in sub-Saharan Africa (including Tanzania) compared to USA [19,20].

That the HIV seropositive patients on HAART (cases) were more likely to have SIL and invasive carcinoma of the cervix than their corresponding controls seems in part, to be related the increased risk behavior for STI including HPV, HSV and in fact HIV among this group as depicted by marital status (singles), number of lifetime sexual partners (multiple) and an early age at first sexual intercourse [21-23]. A previous study showed that inflammatory changes on cervical cytological smears often indicate the presence of STI and masked underlying premalignant disease of the cervix [24].

Due to limitations in resources, the isolation of Chlamydia in cell culture as well as the use of monoclonal antibody to detect Chlamydia elementary bodies could not be achieved in the current study. Nevertheless, our index study indicated that features suggestive of Chlamydia cervicitis were more common among HIV infected patients, which corroborates with several previous reports in which researchers reported that the highest prevalence of *Chlamydia trachomatis *(CT) there was found in HIV infected individuals [25,26]. CT is the most common cause of STI and cytological changes in Pap smears documented [25]. However, a previous study reported that some Chlamydia negative patients had a Pap test which was reported to contain inclusions consistent with Chlamydia infection [27]. The author speculated that the inclusions were probable aggregates of bacteria, cell debris and/or other artifacts thus concluding that the Pap test has low sensitivity compared to Chlamydia culture. Furthermore, follicular cervicitis (FC) also identified in this study is reported to be associated with CT infection [28].

Squamous cell carcinoma was the most predominant type of cancer found among HIV infected patients which would be consistent with a concurrent HPV infection as suggested in our results and would be in agreement with a previous report indicating that HIV alters the natural history of HPV infection, with decreased regression rates and more rapid progression to high grade and invasive lesions, which are refractory to treatment, requiring more stringent intervention and monitoring [5]. However, a previous report suggested a high frequency of adenocarcinoma amongst HIV-infected patients [29] and epidemiological and sample population differences may explain the discrepancy. However, both the current and the previous studies report increasing frequency of amongst those aged ≥ 56 years as expected [29]. Cervical cancer in women younger than 25 years is extremely rare [10]. HIV infected women with cervical cancer before the age 35 years is considered an acquired AIDS-defining illness by the Centers for Diseases Control (CDC) [5,30,31].

The relationship between high parity, OC use and cervical cancer has been suggested and is probably attributed to hormonal factors [29,32].

Loss of wild-type p53 activity is an important event in neoplastic transformation in cervical squamous epithelium and is thought to occur by its degradation by the E6 viral protein of HPV 16 and HPV 18 [33]. On the basis of these mechanisms, clinically relevant p53 immunoreactivity would not be expected in HPV related cervical carcinoma and SIL and this is shown in our current study which also concurs with previous reports suggesting that the expression of p53 is decreased in lesions infected with intermediate and high risk HPV due to the differential ability of HPV E6 proteins to bind to and degrade p53 [33,34]. Thus the frequency, distribution, risk factors and pathogenesis of cervical lesions amongst a selected cohort of Tanzanian women, have been further elucidated by our current study.

## Conclusions

The cytological findings in this study suggest that the frequency of SIL and carcinoma appeared to be higher among HIV-infected women on HAART compared to seronegative controls and as expected increased with age. HIV seropositive patients appeared to present earlier with SIL compared to those HIV seronegative suggesting a role of HIV in altering the natural history of HPV infection and cervical lesions. The absence of p53 immunoreactivity in HPV + lesions is indicative of the ability of HPV E6 proteins to interact with the tumor suppressor gene and pave way for viral-induced oncogenesis in the studied Tanzanian women.

## Competing interests

The authors declare that they have no competing interests.

## Authors' contributions

LMM conceived the study, collected tissue specimen, clinical records, and performed the staining, data analysis as well as contributed to manuscript writing. ARM conceived the study, supervised proposal write-up, taught, performed and supervised the staining, performed microphotography, supervised data analysis, wrote the manuscript, corresponded with editors and publishers and effected all reviewers' and editorial corrections. HAM conceived the study, supervised proposal write-up and data analysis and corrected the manuscript. EEK conceived and supervised the study, procured reagents, taught and supervised the staining and corrected the manuscript. All authors read and approved the final manuscript.
